# Clinical Approach to Acute Recreational Drug Intoxication in the Emergency Setting: A Practical Guide Based on Swiss Experience

**DOI:** 10.3390/toxics13121034

**Published:** 2025-11-29

**Authors:** Patrick Bless, Diane Blaser, Thomas Castelain, Sébastien Pugnale, Vincent Ribordy, Youcef Guechi

**Affiliations:** 1 Department of Emergency Medicine, Fribourg Cantonal Hospital, 1752 Villars-sur-Glâne, Switzerland; thomas.castelain@unifr.ch (T.C.); sebastien.pugnale@unifr.ch (S.P.); vincent.ribordy@h-fr.ch (V.R.); youcef.guechi@h-fr.ch (Y.G.); 2 Faculty of Science and Medicine, University of Fribourg, 20 Avenue de l’Europe, 1700 Fribourg, Switzerland; blaser.diane@gmail.com; 3 Laboratory for Education and Health Promotion, Paris-Sorbonne University, 93430 Villetaneuse, France

**Keywords:** novel psychoactive substances, khat, stimulants, cocaine, opioids, acute intoxication, emergency room

## Abstract

Acute intoxications related to recreational drug use represent an increasing challenge for emergency departments (EDs). Worldwide, more than 600,000 deaths are attributable each year to illicit drug consumption, and in Switzerland approximately 190 deaths related to recreational drug use were reported in 2023. Most patients present after the use of recreational drugs such as stimulants (cocaine and amphetamines), opioids, cannabis or hallucinogens, with stimulants representing the majority of acute presentations in European emergency departments. In recent years, a sharp progression of amphetamine-like substance consumption derived from khat has been observed. Clinical presentations range from agitation, psychosis, seizures, and hyperthermia to respiratory depression, multi-organ failure and cardiac arrest. Emergency physicians are frequently the first to provide care, yet management is often complicated by the coexistence of multiple substances, the emergence of new psychoactive compounds, and the limited availability of toxicological testing in the acute setting. This narrative review summarises the current evidence and recommendations relevant for clinical practice. It is based on a literature search conducted in PubMed, EMBASE, Cochrane Library, but also grey literature such as MonAM, Infodrog and Tox Info Suisse regarding the specific Swiss context. The review highlights the recognition of typical toxidromes: stimulants causing sympathomimetic effects; opioids with mainly respiratory depression; hallucinogens and dissociatives; also, this review presents common pitfalls with drugs commonly encountered. Management emphasises oxygen administration, benzodiazepine sedation for agitation, and specific interventions like naloxone for opioids. Given rising trends in cocaine and novel psychoactive substance use, enhanced understanding of pharmacological profiles and standardised emergency protocols are critical for improving patient outcomes. Although specific treatment may be lacking for most drugs, novel psychoactive drugs pose new challenges due to lack of robust data preventing us from presenting a typical clinical picture and providing standardised care. This synthesis aims to support emergency physicians in the structured and evidence-based management of acute recreational drug intoxications.

## 1. Introduction

According to the WHO, more than 2.6 million deaths worldwide are attributable to alcohol consumption every year, while 600,000 result from psychoactive drug use [1]. Between 2014 and 2017, the European Union Drugs Agency (EUDA) recorded 23,947 emergency room visits for acute recreational drug intoxication in Europe, representing 0.3% of all emergency department consultations [2]. Heroin represented 22% of the substances involved in these cases, followed by cocaine (19%), cannabis (17%) and new psychoactive substances (9%). The overall mortality rate was 0.4%, almost half of which occurred directly in emergency departments. However, Euro-DEN data must be interpreted with caution due to significant geographical variation and limited national coverage. In Switzerland, only three sentinel emergency departments, Basel, Bern, and Lugano, contribute to the Euro-DEN Plus dataset, which limits the generalizability of findings to the national level.

**Figure 1:**
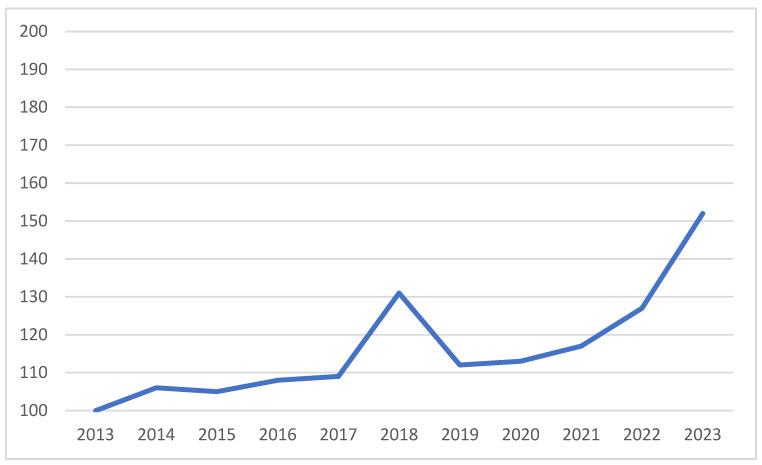
Switzerland (2013–2023), Recreational-drug-related deaths by overdose. Source: Swiss Health Observatory (Obsan), MONAM indicator, Drug-related deaths indicator [5].

**Figure 2:**
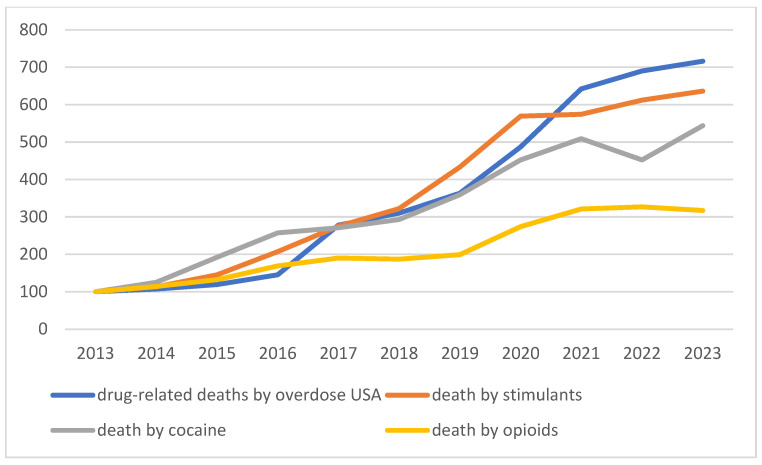
United States (2013–2023), Recreational drug overdose deaths overall and by major substance class (opioids, stimulants, cocaine). Source: National Institute on Drug Abuse (NIDA), Overdose Death Rates [7].

In Switzerland, which has a population of 8.8 million, 137 deaths related to acute recreational drugs intoxication were recorded in 2017, representing a rate of 1.6 per 100,000 inhabitants [3]. Despite a prevention policy and a plan of measures to combat addiction [4], the annual number of deaths associated with the use of psychoactive substances appears to be rising again, reaching an estimated 192 in 2023 [5]. The prevalence of illegal substance use (excluding cannabis) was 8.6% among people aged 15 to 64 in 2022, compared to 3.2% in 2002. Treatment admissions for problematic use varied by substance: between 2013 and 2022, they fell overall for opioids (−31.7%, mainly heroin and morphine), cannabis (−7.9%), and alcohol (−3.7%), but rose sharply for cocaine (+116.9%) [6]. A similar trend was observed in European countries between 2014 and 2017 [2]. In the United States, 105,000 overdose-related deaths were recorded in 2023. Although consumption trends are broadly comparable to those observed in Europe and Switzerland, the situation diverges regarding synthetic opioids, which have experienced a significant epidemic peak since 2014. This is primarily attributed to the substantial presence of illicitly manufactured fentanyl, which is responsible for most deaths [7]. Those trends are illustrated in Figure 1 and Figure 2.

In recent years, new substances have emerged, particularly within the stimulant family. These are mainly synthetic cathinones derived from khat. They are in demand because their effects are similar to those of classic stimulants, such as cocaine, amphetamines and MDMA (3,4-methylenedioxy-*N*-methylamphetamine), but they are more affordable [8,9]. These substances are now widespread and present on the Swiss market. They can be found in many black-market products, often without consumers’ knowledge [10]. As a result, consumers may ingest substances other than those they expect, such as unidentified synthetic derivatives or more concentrated drugs, thinking they are taking a familiar product.

This article aims to provide emergency physicians with a practical, structured framework for assessing and managing acute intoxications linked to the most common recreational drugs by combining international data and clinical experience. The most frequently encountered substances can be classified into four main categories: stimulants, opioids, hallucinogens and dissociative psychoactive substances. For each category, we present the key mechanisms of action, characteristic clinical features, and validated therapeutic strategies for the emergency context.

## 2. Materials and Methods

A narrative review approach was used. Because this work is not a systematic review, it does not follow the PRISMA-S guidelines. However, the search strategy was conducted transparently and structured according to good-practice principles. The substances covered were selected according to three main criteria:

1. Prevalence of use, based on national epidemiological indicators from MonAM (FOPH) and Addiction Suisse reports;
2. Frequency of presentation to emergency departments, based on the authors’ clinical experience and local data; and
3. Potential danger in the acute phase, defined as the risk of life-threatening complications or the need for specific treatment.

We searched PubMed, Embase, and the Cochrane Library for publications between January 2000 and March 2025 using combinations of the keywords “recreational drugs”, “acute intoxication”, “emergency department”, “toxidrome”, and “management”.

Clinical guidelines, systematic reviews, observational studies, and expert consensus documents relevant to the emergency care of acute recreational drug intoxications were eligible. Articles in English, French, or German were considered. Experimental studies and single case reports were excluded unless they provided clinically applicable information. Titles and abstracts were screened by two authors independently (P. Bless and Y. Guechi), and full texts were assessed for relevance; disagreements were resolved by consensus. Grey literature from Swiss national sources (MonAM, Infodrog, Tox Info Suisse) was also reviewed to capture epidemiological data and local recommendations. Whenever possible, recommendations were classified according to their origin (toxicology society guideline, systematic review, expert consensus). No formal quality appraisal was performed, consistent with the narrative design of the review.

This review aimed to offer a concise and practice-oriented update for practitioners, who often face these situations alone, based on the available data and the authors’ hospital experience, rather than providing an exhaustive assessment of the literature or a quantitative synthesis.

## 3. Results

The main findings of this review are summarised in Table 1, which outlines the most common recreational drugs, their characteristic clinical features, diagnostic pitfalls, and recommended emergency management strategies. This table serves as an overview and reference framework, while the following subsections provide a more detailed description for each drug category. With a concern for synthesising and strategically grouping information, all complications inherent to recreational drugs intoxication but shared by several substances are listed in Table 2.

### 3.1. Stimulants

#### 3.1.1. Cocaine

Cocaine is a naturally occurring alkaloid found in the leaves of the Erythroxylum coca plant, which is mainly cultivated in the Andes. It has been used by Inca populations for over three millennia for its psychostimulant properties and was exploited by native workers under Spanish colonisation for utilitarian, recreational and ritual purposes to improve their stamina. From the 1850s onwards, a German pharmacist isolated its active ingredient and produced it as an elixir, leading to widespread medical and commercial distribution, particularly in the form of certain beverages (such as Mariani wine and Coca-Cola^®^). The first regulations appeared in the United States as early as 1906 [11]. Today, cocaine is authorised in the United States for local anaesthesia in ENT at concentrations of 4% and 10% [12].

Cocaine is used more frequently by men than women (6.2% versus 2.4%), with an overall prevalence of consumption of 8.4% by the age of 30. Cocaine use rose from 2.2% of the general population in 2002 to 6.2% in 2022 in Switzerland [6,13]. It is generally taken intranasally, smoked or injected. Due to its rapid absorption through the airways, it is primarily consumed by inhalation or smoking [14]. It can also be found in tablets sold as amphetamines [10] without the knowledge of the consumer.

Cocaine blocks voltage-dependent sodium channels, producing a local anaesthetic effect and a type I antiarrhythmic effect. However, in the context of intoxication, this effect is counterbalanced by intense adrenergic stimulation, making cocaine overall pro-arrhythmogenic. It inhibits the presynaptic reuptake of noradrenaline, dopamine and serotonin, thereby inducing powerful stimulation of the central nervous system and a pronounced sympathomimetic effect [14,15].

Acute complications include tachyarrhythmia, prolonged QTc and QRS intervals, hypertension and chest pain related to vasospasm. Up to 25% of myocardial infarctions in people aged 18–45 are attributed to acute cocaine use [14,16,17]. Vasospasm affects the whole body, inducing mesenteric ischemia [18], pulmonary damage, and damage to other organs, as well as hyperthermia and its consequences, such as rhabdomyolysis and disseminated intravascular coagulation (DIC), due to reduced peripheral circulation [15]. Neurological consequences include ischemic strokes and intracranial haemorrhages, as well as disturbances to synaptic conduction, resulting in epileptic seizures, hyperthermia and mydriasis. Psychiatric consequences include psychotic episodes [14,19].

The necessary paraclinical investigations include an ECG, a simple blood count, renal function, electrolytes (sodium, potassium and calcium), troponin, CK and CK-MB, clotting time, blood gases with lactates and bicarbonates, blood glucose, monitoring of body temperature, and a pregnancy test for women in childbearing age. A CT scan or lumbar puncture with xanthochromia testing may be necessary to investigate intracranial haemorrhage if clinical signs suggest this. This list is not exhaustive and must be adapted to the patient’s clinical presentation.

Initial treatment involves targeting an oxygen saturation of at least 93% to ensure sufficient oxygenation of tissues in the context of systemic vasoconstriction. Benzodiazepines are indicated in case of agitation, seizures, sympathomimetic symptoms and hyperthermia [20] prior to the specific management of intoxication consequences (DIC, ischemic or haemorrhagic stroke, rhabdomyolysis, etc.). Midazolam is preferred due to its ease of intravenous or intramuscular administration, rapid onset of action, and short half-life. Midazolam is titrated at 2–4 mg IV every 15 min until the desired level of sedation is reached. If intubation is required, succinylcholine should be avoided as it is metabolised by plasma cholinesterase, an enzyme that is also involved in cocaine metabolism. Their interaction could prolong the myorelaxant effect or intoxication [21]. For the management of cardiac complications, the addition of a cardio selective beta-blocker such as esmolol (0.5–1 mg/kg IV bolus, then 50–200 µg/kg/min continuous infusion) may be considered. However, due to the risk of increased α-adrenergic vasoconstriction, combining it with a vasodilator such as nitroglycerin (2–10 mg/h IV) is recommended to prevent hemodynamic side effects. Certainly, active cooling must be initiated in case of hyperthermia, using external methods such as ice packs, cooling blankets or evaporative techniques.

Finally, dexmedetomidine (a selective α_2_-receptor agonist with a central sympatholytic effect) may be considered in the event of severe neuropsychiatric manifestations (administered intravenously at a rate of 0.7–1.4 µg/kg/h).

Specific complications of cocaine intoxication mainly involve pulmonary complications (crack lung, pneumothorax and pneumomediastina, alveolar haemorrhage), coronary syndrome, ischaemic stroke, haemorrhagic stroke and mesenteric ischaemia.

Crack lung, a syndrome of haemorrhagic alveolitis associated with inhaled cocaine, is investigated with chest radiograph looking for infiltrates in a patient presenting acute lung injury with chest pain and haemoptysis, fever and/or bronchospasm. Management relies on supportive measures and control of coagulation. The same investigations and management apply for alveolar haemorrhage in the context of cocaine use.

Pneumothorax and pneumomediastinum should be investigated using chest radiograph or thoracic CT scan in case of doubt. Management depends on the severity, ranging from supportive care to surgical intervention.

Coronary syndrome, ischaemic stroke and mesenteric ischaemia are treated with benzodiazepines and, in severe cases, vasodilators such as nitroglycerin, as suggested above. Treatment should counteract vasospasm and restore perfusion. If this fails, it is necessary to perform a coronary angiogram, a contrast-enhanced brain CT scan or a contrast-enhanced abdominal CT scan to look for possible occlusion and initiate standard treatments for these conditions.

The management of haemorrhagic stroke is based on strict blood pressure control with systolic blood pressure < 140 mmHg, mean blood pressure < 90 mmHg, control of coagulation and, in the event of intracerebral mass effect, craniotomy.

The duration of the effects of cocaine varies from 15 min (inhaled) to 90 min (intranasal). Some studies have identified slow metabolizers who take up to 180 min to eliminate the substance and its acute effects. Close monitoring is necessary during this period and may be prolonged for up to 48 h in the intensive care unit in cases of high dosage and specifically depending on any vascular complications identified (e.g., STEMI, stroke, intestinal ischemia, etc.).

#### 3.1.2. Amphetamines (Including Methamphetamine and MDMA) and Synthetic Cathinones

Amphetamines were first synthesised in 1887 by the Romanian chemist Lazăr Edeleanu under the name phenylisopropylamine [22]. After being forgotten, the molecule reappeared in the 20th century, being used as early as 1914 as a “truth serum”. It was then widely administered to soldiers during the Second World War to improve endurance and alertness [23]. However, the undesirable effects of these substances were widely publicised, notably following the death of cyclist Tom Simpson in 1967. They were classified as psychotropic substances in 1971 and banned by the International Olympic Committee and the World Health Organisation in 1980. Today, amphetamines are used to treat attention deficit hyperactivity disorder [24], as a short-term treatment for obesity (as an appetite suppressant), and off label to treat narcolepsy. MDMA (3,4-methylenedioxy-methamphetamine) was synthesised in 1914 and initially developed as an anorectic [25]. Cathinones are derived from khat (Catha edulis), a shrub that is traditionally consumed in the Horn of Africa and the Arabian Peninsula for its mildly euphoric effects. Their chemical transformation has led to a massive boom in synthetic cathinones since the 2000s [26]. They are easy to produce and are often marketed in tablet or powder form. Users frequently consume them without knowing their true nature [10]. They are consumed in tablet form but can also be smoked or injected.

In 2012, 5.1% of people surveyed aged 15–39 said they had used a stimulant drug (amphetamine, methamphetamine or MDMA) at least once [27]. According to the Swiss Monitoring System for Addiction and Non-Communicable Diseases (MonAM), this figure is expected to rise to 10.8% by 2022 when all stimulant drugs are considered, excluding cocaine [28].

Amphetamines enter the presynaptic terminal by passive diffusion, inducing the massive release of dopamine and noradrenaline via the lysis of synaptic vesicles independently of exocytosis [29,30]. Some amphetamines also inhibit monoamine oxidase (MAOIs), thereby prolonging the half-life of catecholamines and serotonin. MDMA has a direct effect on serotonin receptors, which explains its psychostimulant and hallucinogenic properties [31].

The clinical presentation resembles a sympathomimetic toxidrome, characterised by tachycardia, hypertension, mydriasis, hyperthermia and agitation, and sometimes hallucinations. Hyperthermia is central to the toxidrome and is due to excess catecholamines rather than infection. Severe complications may arise, including myocardial ischemia, ischemic stroke due to vasospasm, rhabdomyolysis, acute renal failure, metabolic acidosis, and disseminated intravascular coagulation (DIC), sometimes requiring intensive care [32,33,34]. Severe hypoperfusion, which is often underestimated in young patients, can lead to mesenteric or myocardial infarction or distributive or cardiogenic shock [35]. Synthetic cathinones are particularly associated with extreme agitation, paranoid delusions, and acute toxic psychosis [36,37].

The necessary paraclinical investigations include an ECG, a simple blood count, renal function, electrolytes (sodium, potassium and calcium), troponins, CK and CK-MB, ALAT and ASAT, clotting time, blood gases with lactates and bicarbonates, blood glucose, and a pregnancy test for women in childbearing age. A CT scan or lumbar puncture with xanthochromia testing may be necessary to investigate intracranial haemorrhage if clinical signs suggest this. This list is not exhaustive and must be adapted to the patient’s clinical presentation.

Moderate cases usually require clinical monitoring in a supervised environment. In cases of severe intoxication, administering oxygen (SpO_2_ ≥ 93%) is essential. Midazolam is recommended in cases of agitation (2 to 4 mg IV every 15 min, depending on titration) due to its rapid effect, short half-life and multiple routes of administration. If intubation is required, however, succinylcholine is contraindicated due to the risk of rhabdomyolysis-induced hyperkalaemia. In cases of severe cardiovascular dysfunction, caution should be exercised when using a cardioselective beta-blocker (such as esmolol: 0.5–1 mg/kg IV bolus followed by a 50–200 µg/kg/min infusion). Hyperthermia of ≥40 °C requires active cooling combined with deep sedation and muscle relaxation to reduce metabolic heat production. In cases of multivisceral failure or severe electrolyte disorders, intensive care management involving early recourse to extracorporeal purification is necessary [15,38,39].

Specific complications of amphetamine intoxication include hyperthermia, seizures and status epilepticus, cerebral oedema due to rupture of the blood–brain barrier, probably induced by oxidative stress, hypertension and hyperthermia, as well as hyponatraemia, and renal failure followed by multiple organ failure. The three first cited and their management are detailed in Table 2.

Renal failure is treated as described above and requires urgent transfer to intensive care for continuous renal replacement therapy.

Amphetamines represent such a wide variety of molecules that it is difficult to define a precise duration of action. We will assume a duration of action that can vary mainly from 4 to 6 h, with average monitoring equivalent to this duration of action. In cases involving specific and severe complications, intensive care monitoring may be required for several days or even weeks.

### 3.2. Opioids

The use of opium dates back to the Sumerian civilization around 3000 BC, when it was sought after for its sedative and analgesic properties. In ancient Greece, its therapeutic properties were recognised, but the first cases of abuse were also reported. As early as 1729, the Chinese emperor banned its import due to its harmful effects, but the trade was revived by European colonial powers, notably during the Opium Wars of the 19th century (1839–1842).

Heroin (diacetylmorphine), a derivative of morphine, was synthesised in the late 19th century by an English chemist as an antitussive and a morphine substitute. It was widely used by the military during major conflicts in the 19th and 20th centuries, such as the American Civil War and the Vietnam War, where high rates of addiction were observed (up to 20% of combatants were heroin addicts). Despite international regulatory efforts initiated by the Hague Convention (1912) and relayed by the League of Nations, non-medical use of heroin persisted until it was gradually prohibited in Europe from 1931 and in the United States in 1956 [40,41,42].

In Switzerland, despite a fall in arrests for narcotics offences and opioid agonist prescriptions, the situation remains concerning. Between 2000 and 2019, calls to Tox Info Suisse regarding opioid intoxication increased by 177%, alongside a significant rise in the sales of strong opioids (+669.6%) and weak opioids (+25.2%) [43,44,45]. The most frequently involved opioids include tramadol and oxycodone, followed by morphine, codeine, buprenorphine, and fentanyl. This trend suggests an epidemiological shift towards prescription-only use, particularly among elderly patients with chronic pain [46]. Nevertheless, some people continue to inject or inhale opioids for recreational purposes. Heroin use appears to be stable with 0.7% of the general population declaring (voluntarily and anonymously) consuming some in 2002 against 0,8% in 2022 [6,47].

From a pharmacological perspective, opioids primarily exert their effects via the activation of μ (mu), δ (delta) and κ (kappa) opioid receptors. These receptors are members of the G protein-coupled receptor (GPCR) family. These receptors are distributed throughout the central nervous system and various peripheral tissues.

The μ receptor, the main target of most opioids (including morphine, heroin and fentanyl), is responsible for respiratory depression by reducing the sensitivity of the bulbar centres to carbon dioxide (CO_2_) and hypoxia. It is also responsible for central analgesic effects, euphoria, sedation and miosis (pupil constriction) [48,49]. Peripheral activation of this receptor can also induce bradycardia and hypotension, notably through reduced sympathetic tone and vagal activation. It reduces the activity of the longitudinal smooth muscle while increasing that of the circular layer, leading to constipation and urinary retention.

δ receptors, which are less targeted by conventional opioids, contribute to the analgesic effect and regulate neurotransmitter release. Their activation is also associated with pro-convulsant effects and alterations in cardiac rhythm, including prolongation of the QTc interval and an increased risk of torsade de pointes under certain conditions [50,51].

κ receptors are mainly involved in spinal analgesia and have side effects such as miosis, sedation, and increased diuresis. The latter is mediated by the inhibition of vasopressin secretion.

Additionally, some opioids stimulate histamine release via mast cell degranulation, resulting in adverse effects such as pruritus, facial erythema (flushing) and pseudoallergic reactions, independent of an immunological mechanism.

Finally, loperamide, a µ-agonist commonly used as an antidiarrheal, is a special case. Under normal conditions, it does not penetrate the central nervous system due to the combined action of P-glycoproteins (P-gp) and hepatic metabolism via CYP3A4, which limits its systemic bioavailability. However, massive overdose or association with enzyme inhibitors (e.g., ketoconazole) can bypass these protective mechanisms, allowing loperamide to cross the blood–brain barrier and induce respiratory depression and sedation comparable to that of central opioids. Furthermore, at high doses, loperamide has been associated with severe ventricular rhythm disturbances, including QT prolongation, torsade de pointes, and cardiac arrest.

The necessary paraclinical investigations include, in particular: an ECG, a simple blood count, renal function, blood gases, blood sugar levels, and a pregnancy test for women of childbearing age.

Initial management focuses on maintaining vital functions, with the early administration of oxygen being key to correcting hypoxemia induced by respiratory depression. A target saturation of at least 93% is recommended to ensure adequate tissue oxygenation, particularly in cases of bradypnea or central apnoea. The etiological treatment is based on naloxone, which is a competitive opioid receptor antagonist with a high affinity for the µ receptor. It rapidly reverses opioid-induced ventilatory depression.

The modalities of administration are as follows:

1. Intravenous (IV): 0.4 mg every two to three minutes until a clinical response is obtained or a maximum cumulative dose of 10 mg is reached.
2. Intramuscular (IM): 2 mg as a single dose, useful in pre-hospital settings or if venous access is difficult.
3. Intranasal (IN): 4 mg per unilateral spray. Suitable for self-administration or by first responders.
4. If a favourable response is observed, a continuous IV infusion of naloxone may be necessary, starting at 0.1–0.6 mg/h and adjusted according to the pharmacokinetics of the substance involved, the severity of intoxication, and the clinical course. It is important to note that highly potent synthetic opioids such as fentanyl and its analogues (e.g., carfentanil and sufentanil) have a much greater affinity for the µ receptor. This often justifies higher doses of the antagonist or prolonged treatment, sometimes for more than 12 h.
5. If there is no clinical response to a 10 mg IV dose of naloxone, the diagnosis should be reassessed with particular attention to mixed intoxication (e.g., benzodiazepines, alcohol or other central nervous system depressants) or an alternative neurological cause.
6. Cardiovascular complications should be managed in accordance with ACLS^®^ protocols, particularly in the following cases:
  - Torsade de pointes: administer IV magnesium sulphate.
  - QTc prolongation or hemodynamic instability: short-acting, cardioselective beta-blockers (e.g., esmolol).

In cases of convulsions, which are often related to the ingestion of atypical opioids (e.g., tramadol or meperidine) or abrupt withdrawal, benzodiazepines are the primary treatment. Slow titration is essential to avoid an increase in respiratory depression caused by the drugs working together, particularly in cases of poly-intoxication.

Specific complication of opioid intoxication (including naloxone) is pulmonary oedema with acute respiratory distress syndrome (ARDS). The pathophysiology of pulmonary oedema is unclear, but in the case of pharmacological reversal, it appears to be linked to the arrival of large quantities of catecholamines during reversal with naloxone in the presence of high PCO2, which induces increased afterload and consequent oedema. Pulmonary oedema is managed with respiratory support using positive pressure and intubation if the patient’s level of consciousness does not allow non-invasive ventilation. Pulmonary oedema is subsequently self-resolving.

Patients must be continuously monitored in a supervised environment and managed for metabolic, ventilatory and neurological complications. Intensive care management is recommended in the event of clinical relapse or the need for high cumulative doses of naloxone. The duration of action of opioids is determined by the specific opioid and its formulation, but monitoring after opioid overdose and naloxone administration should be guided primarily by clinical symptoms rather than a fixed time interval. The most critical period is after the effect of naloxone has worn off, as the duration of action of naloxone (typically 30–90 min) is often shorter than that of many opioids, especially long-acting formulations, creating a risk for recurrent respiratory depression once naloxone is metabolised [52,53,54,55]. These patients should be transferred to the intensive care unit for long-term care.

### 3.3. Hallucinogens/Psychedelics

The Swiss Monitoring System for Addictions and Non-Communicable Diseases (MonAM) reveals a gradual increase in the use of hallucinogenic substances. However, specific data remains limited for certain molecules, such as LSD (lysergic acid diethylamide), new psychoactive substances (NPS), and ketamine [56]. This trend, observed in Switzerland, is part of a global phenomenon involving the emergence of synthetic psychoactive substances that are often undetectable by standard tests and are regularly modified to evade legal restrictions.

#### 3.3.1. LSD and Lysergamide-Type NPS

LSD was synthesised in 1938 by Albert Hofmann from lysergic acid, which is derived from ergot. It was first studied for its vasodilatory properties. The accidental discovery of its psychedelic properties in 1943 led to its use in experimental psycholytic therapies and, subsequently, its misuse in psychological manipulation programmes (e.g., Project MK-Ultra, CIA). LSD was gradually banned beginning in the 1960s [57,58]. Hofmann also isolated psilocybin, the active ingredient in hallucinogenic mushrooms that are distributed around the globe. This marked the beginning of contemporary research into hallucinogenic tryptamines.

According to a Swiss national survey, 3% of the adult population claims to have experimented LSD, with consumption concentrated among 15–34-year-olds [59].

Although the term “novel psychoactive substances” includes a very wide variety of molecules with heterogeneous structures and effects (tryptamines, phenethylamines, and piperazines), this section focuses only on those producing hallucinogenic or psychedelic effects similar to LSD, mainly certain tryptamines and phenethylamines [60,61,62,63]. But their composition changing frequently, it makes it very challenging to detect and quantify them.

From a clinical perspective, these drugs present similar symptoms to LSD intoxication, including visual and auditory hallucinations, derealization, disinhibition, paranoid ideation, mental confusion, delusional episodes, and extreme agitation. These symptoms can lead to risky behaviours such as self-mutilation, running away, jumping from windows, accidents, and aggression [64,65].

Somatic complications include hyperthermia greater than 40 °C, which is not linked to hypothalamic dysregulation, but rather to central sympathetic activation, electrolyte disorders, rhabdomyolysis, and acute renal failure. There is a rare but serious risk of cerebral oedema or multi-organ failure syndrome. Serotonin syndrome, often associated with certain NSPs or drug interaction, may also occur. If left untreated, it can progress to severe hyperthermia, convulsions, lactic acidosis, or multivisceral failure. Direct mortality is low, but deaths indirectly linked to substance-induced behaviour are well documented, particularly in connection with LSD or hallucinogenic phenethylamines (e.g., NBOMe, 2C-B) [65,66].

The necessary paraclinical investigations include an ECG, a simple blood count, renal function, electrolytes (sodium, potassium and calcium), CK, blood gases, lactates and bicarbonates, blood glucose, and a pregnancy test for women of childbearing age.

Treatment is based on managing symptoms tailored to clinical severity. Given how easy it is to manufacture NPS using everyday products, and given that the consequences are still poorly understood, it is extremely difficult to propose a standardised approach. A general approach that can be applied to all patients can be identified, as outlined below, but it is important for clinicians to remain alert to clinical variations that have not yet been identified.

Management includes the following:

1. Avoidance of any stimulation (keep them in a quiet room).
2. Attempt to de-escalate in the event of psychomotor agitation.
3. Sedation with benzodiazepines (e.g., midazolam, 1–2 mg IV) is the first-line treatment.
4. Body temperature monitoring, IV rehydration, and external cooling if necessary.
5. Avoid serotonergic antiemetics (e.g., ondansetron); prefer non-5-HT3 alternatives. Severe cases:
6. Intensive care hospitalisation.
7. Active cooling (e.g., cooling blankets or cold baths).
8. Respiratory or hemodynamic support, if necessary.
9. Cyproheptadine (a 5-HT2A antagonist) may be considered if serotonin syndrome is confirmed. Due to the lack of an IV formulation, its use is limited in the acute setting, but it can be administered via nasogastric tube in a sedated patient. The recommended initial dose is 12 mg orally, followed by 2 mg every two hours. The maximum dose is 32 mg per 24 h. This molecule is, however, not available in all countries.

Specific complications of LSD and lysergamide-type NPS intoxication include hyperthermia and hyponatraemic cerebral oedema. The management of these complications is detailed in Table 2.

The peak effect of LSD occurs approximately 2 to 4 h after ingestion, and the effects can last up to 12 h after ingestion. Monitored supervision is necessary for at least the first 6 h and must be adapted to clinical developments. Regarding lysegamide-type NPS, their compositions are too variable to identify a typical duration of action. Therefore, the clinician must adapt to the patient’s clinical presentation.

#### 3.3.2. Cannabinoids

Although cannabinoids are among the most widely used psychoactive substances worldwide, their pharmacology and acute toxicity remain only partially understood [67,68,69,70]. The term encompasses a wide spectrum of compounds, from natural cannabis (Δ^9^-tetrahydrocannabinol, THC) to numerous synthetic analogues that continue to emerge on the recreational market [71,72]. The growing chemical diversity of these substances, including modified CBD derivatives, makes both detection and clinical interpretation increasingly complex. Synthetic cannabinoids were first synthesised in the 1990s by John W. Huffman and colleagues as research ligands for cannabinoid receptors. They were later diverted for recreational purposes due to their extreme potency and ability to evade drug regulations. Production and online dissemination increased markedly during the SARS-CoV-2 pandemic, when access to other drugs declined. In Switzerland, as in many European countries, these compounds were officially classified as narcotics in 2023. Since then, the continuous development and marketing of new derivatives have outpaced regulatory control. Self-reported consumption of cannabinoid products has steadily increased, from approximately 20% of adults in 2002 to over 32% in 2022 [6]. Polysubstance use, especially with alcohol, stimulants, or benzodiazepines, is frequent and further complicates the clinical presentation.

##### Natural Cannabinoids (THC)

THC acts as a partial agonist at CB1 and CB2 receptors. Its psychoactive effects (relaxation, euphoria, altered perception) are typically short-lived and mild [68,70]. The most common emergency presentations involve anxiety, tachycardia, dizziness, or transient psychotic reactions. Severity varies widely depending on dose, route of use, and individual tolerance. Chronic heavy consumption may lead to cannabinoid hyperemesis syndrome, characterised by cyclical vomiting relieved by hot showers.

Rare complications related to THC include acute coronary events [73,74], asthma exacerbations, pneumothorax [75,76], and transient ischemic attacks.

Acute coronary syndrome has been described after cannabis use, mostly in young adults without prior cardiovascular disease; the mechanism likely involves increased myocardial oxygen demand and sympathetic stimulation rather than coronary vasospasm.

Pneumothorax and pneumomediastinum are attributed to deep inhalation and Valsalva-type manoeuvres during smoking; treatment relies on oxygen therapy and pleural decompression if needed.

Asthma exacerbations arise from airway irritation due to smoke and combustion products, requiring management according to standard guidelines (oxygen, bronchodilators, corticosteroids, hydration ± magnesium sulphate).

Management of THC intoxication is supportive. A calm environment, reassurance, and observation are generally sufficient. Benzodiazepines (e.g., midazolam 1–2 mg IV) may be administered for agitation or panic. Most cases resolve spontaneously within a few hours.

##### Synthetic Cannabinoids

By contrast, synthetic cannabinoids are full agonists at cannabinoid receptors and can be several hundred times more potent than THC. Their composition and concentration vary considerably between products, leading to unpredictable toxicity [77,78,79]. Typical emergency presentations include agitation, hallucinations, psychosis, seizures, tachycardia, hypertension, and arrhythmias. Severe forms may feature hyperthermia with rhabdomyolysis, acute kidney injury [80,81,82,83], and metabolic acidosis.

The principal complications associated with synthetic cannabinoids exposure are seizures or status epilepticus, hyperthermia-related rhabdomyolysis, and cardiovascular instability (tachyarrhythmias, hypertension, or, rarely, myocardial ischemia).

Seizures are managed with benzodiazepines (midazolam IV 2 mg or IM 10 mg); second doses after 5 min if IV; if refractory switch to status epilepticus protocol (see Table 2).

Hyperthermia warrants active cooling, sedation with benzodiazepines if agitation and orotracheal intubation with neuromuscular blockade if refractory; aggressive IV hydration helps prevent renal failure from rhabdomyolysis.

Cardiac rhythm disorders and severe hypertension necessitate continuous monitoring and symptomatic treatment; beta-blockers should be avoided unless under expert supervision.

Stroke (ischemic or haemorrhagic) has occasionally been reported but remains of uncertain causality [84,85]. As the pathophysiology is unclear, they should be managed like any ischemic or haemorrhagic stroke.

Because onset and duration of symptoms are variable, prolonged observation or ICU admission may be required, particularly after exposure to high potency synthetic analogues (such as JWH-018 or AMB-FUBINACA) [86,87].

## 4. Implications for Practice and Training

The practical implications highlighted in this review are the following: the importance of a structured approach based on the ABCDE scheme familiar to emergency physicians and focused on toxidromes (sympathomimetic, opioid, hallucinogenic), enabling rapid and targeted treatment; the early administration of oxygen therapy, benzodiazepines for stimulants or agitation, and naloxone for opioids; recognition of the pitfalls associated with new psychoactive substances; and the importance of recognising co-intoxications as early as possible. Table 1 proposes a standardised approach to intoxicated patients once the underlying substances or syndrome have been identified. Given the significant increase in psychostimulant intoxications, it is necessary to strengthen knowledge in this area within emergency departments. Furthermore, it is important to recognise that voluntary intoxication does not justify different medical treatment.

The decontamination of a patient who has recently ingested a recreational drug is not addressed in this document. Activated charcoal decontamination is only effective within one hour of ingestion, and most patients arrive at the ED when the effects of intoxication are already advanced, well after absorption has occurred. Furthermore, administration of activated charcoal carries a significant risk of aspiration, especially in agitated or sedated patients, and should therefore only be considered when airway protection is ensured. Other decontamination methods, such as gastric lavage, are not recommended in poisoning management due to the absence of demonstrated benefit and the potential for complications.

The diagnostic tests available in Swiss emergency rooms remain limited. Substance screening is performed using the KIMS (kinetic interactions of microparticles in solution) method on urine samples. Qualitative and semi-quantitative analyses are available for benzoylecgonine (the main metabolite of cocaine), most known amphetamines and methamphetamines (note that the constant growth in the variety of these molecules means that results cannot be guaranteed), morphine and its metabolites (allowing the presence of opioids to be detected without specifying the substance), barbiturates, benzodiazepines and cannabis metabolites. However, these screening tests do not exclude specific substances, as they are subject to both false negatives and false positives depending on the assay used. Comprehensive and specific analytical methods are available but are rarely performed due to their high cost and delayed turnaround time, which usually prevents them from influencing acute management. Consequently, initial treatment in the emergency department should primarily focus on the patient’s clinical condition and associated complications.

This work is based on an extensive synthesis of clinical and toxicological evidence, complemented by practical experience from Swiss emergency departments. Although it does not follow the methodology of a systematic review, it integrates data from a broad range of peer-reviewed studies and toxicology sources, providing a clinically relevant overview. The main limitation lies in the heterogeneity of available data and the frequent occurrence of mixed intoxications involving several substances, which can result in complex or atypical clinical presentations that differ from the simplified presentations described in Table 1. Moreover, illegal drugs may contain adulterants or undeclared substances, and toxicological analytics are frequently unavailable. Therefore, epidemiological data and observed trends likely only approximate the real situation.

## 5. Conclusions

This review provides a structured and practice-oriented synthesis of the clinical management of acute recreational drug intoxications. It highlights the need of toxidrome-based approach combined with the ABCDE framework to enable rapid and targeted intervention in the emergency settings. It also underlines that apart from opioids, specific antidote treatments are rare, whereas rapid sedation with benzodiazepines play a key role in the management of sympathomimetic syndromes. Most treatments remain supportive, and at least for the time being, must be based primarily on clinical assessment rather than toxicological screenings, as these tests are often delayed or unavailable in an emergency setting.

While opioid, and cannabis use are declining, cocaine, amphetamine, and new psychoactive substance use is rising sharply. These last substances represent a big challenge for researcher and clinician due to their chemical variability and constant evolution.

The frequent combination of multiple substances and the rapid emergence of new unregulated analogues make clinical management increasingly complex and highlight the urgent need for coordinated toxicovigilance and adaptive regulatory frameworks.

Given these developments, future research should focus on developing evidence-based treatment algorithms for specific NPS-related presentations and strengthening clinician training in recognising evolving toxidromes. Collaborative surveillance networks integrating clinical and toxicological data may also help anticipate new trends and guide public health responses.

## Figures and Tables

**Table 1:** Summary of clinical presentations and emergency management of acute recreational drug intoxications. Abbreviations: ABG = Arterial Blood Gas, DIC = Disseminated Intravascular Coagulation IV = Intravenous, IM = Intramuscular, IN = Intranasal, HR = Heart Rate, HTN = Hypertension, ICU = Intensive Care Unit, ACLS = Advanced Cardiac Life Support, NPS = New Psychoactive Substances, NTG = Nitroglycerin, PO = Per os (oral administration), SBP = Systolic Blood Pressure, HD = haemodialysis, AKI = Acute Kidney Injury.

Drug	Typical Clinical Features	Pitfalls	Treatment
Cocaine	Tachycardia, hypertension, arrhythmias, chest pain, myocardial infarction, hyperthermia (rhabdomyolysis, DIC), mydriasis, ischemic strokes, intracranial haemorrhages, seizures, agitation, psychotic episodes.	Risk of generalised vasospasm (coronary, cerebral, mesenteric) Avoid succinylcholine (metabolised by plasma cholinesterase, also involved in cocaine metabolism) Avoid β-blockers alone (α-mediated vasoconstriction risk).	O_2_ (SpO_2_ ≥ 93%) Benzodiazepine as sympatholytic (midazolam IV 2–4 mg titrated every 15 min) to target SBP < 140 mmHg and HR < 110 ppm Cardioselective β-blocker + vasodilator (esmolol IV 0.5–1 mg/kg bolus, then 50–200 µg/kg/min continuous infusion) + NTG IV 2–10 mg/h) to reach HR and SBP mentioned above For seizure and status epilepticus, see Table 2 Dexmedetomidine (IV 0.7–1.4 µg/kg/h) if neuropsychiatric symptoms
Amphetamines and cathinones	Sympathomimetic toxidrome: tachycardia, hypertension, myocardial ischemia, mydriasis, agitation, hallucinations, psychosis, hyperthermia (rhabdomyolysis, DIC), seizures, ischemic stroke.	Hyperthermia = life-threatening. Do not confuse with infection. Avoid succinylcholine (risk of hyperkalaemia in rhabdomyolysis) Severe hypoperfusion often underestimated	O_2_ (SpO_2_ ≥ 93%) Cardioselective beta-blocker + vasodilator if benzodiazepines insufficient (see cocaine treatment above) For seizure and status epilepticus, see Table 2 IV fluids, correct electrolytes ICU if multiorgan failure (e.g., HD in case of AKI)
Opioids (heroin, morphine, fentanyl, tramadol, oxycodone, loperamide)	Respiratory depression, bradypnea, coma, miosis, bradycardia, hypotension, constipation, seizures (tramadol), pseudoallergic reactions.	Synthetic opioids (fentanyl) often require higher and prolonged naloxone dosing Risk of cardiovascular complications	O_2_ (SpO_2_ ≥ 93%) Naloxone (IV 0.4 mg q2–3 min, max 10 mg; IM 2 mg; IN 4 mg), continuous infusion if needed, starting at 0.1–0.6 mg/h ACLS for arrhythmias: IV magnesium sulphate for Torsade de pointes, beta-blocker (e.g., esmolol if QTc prolongation or hemodynamic instability) For seizure and status epilepticus, see Table 2
Hallucinogens (LSD, psilocybin, lysergamide-type NPS)	Visual/auditory hallucinations, agitation, delirium, panic attacks, serotonin syndrome (HTN, hyperthermia, seizures), hyponatraemic cerebral oedema, metabolic acidosis and acute renal failure.	Risk of dangerous behaviours (self-harm, fleeing, defenestration) NPS unpredictable (variable composition)	Avoidance of any stimulation and de-escalation if possible Benzodiazepines (midazolam IV 1–2 mg) for sedation and sympatholytic effects if de-escalation not effective For seizures, status epilepticus, hyperthermia and hyponatraemic cerebral oedema, see Table 2 Avoid serotonergic antiemetics (e.g., ondansetron), prefer non-5-HT3 alternatives
Cannabinoids (THC, synthetics)	Asthma exacerbation, tachycardia, thoracic pain, seizures (synthetics), hallucinations, seizures (synthetics), hyperthermia (synthetics), hyperemesis, acute anxiety, panic attacks, agitation.	Synthetic cannabinoids are more toxic and unpredictable than natural THC.	Symptomatic management, Monitoring Benzodiazepines if agitation/anxiety Asses any asthma attack, thoracic pain and hemi-syndrome like any other patient For seizure, status epilepticus and hyperthermia, see Table 2 Psychological support

**Table 2:** Summary of the Management of Common Complications of Acute Recreational Drug Intoxications (Abbreviations: DIC = Disseminated Intravascular Coagulation, IV = Intravenous, IM = Intramuscular, HR = Heart Rate, SBP = Systolic Blood Pressure).

Complication	Management	Additional Measures/Remarks
Hyperthermia	Active cooling: ice packs, evaporative cooling Sedation with benzodiazepines if agitation In case of refractory severe hyperthermia, neuromuscular blockade and IOT should be considered	Close monitoring of body temperature Preventive management helps reducing incidence of cerebral oedema.
Seizures	Benzodiazepine (midazolam IV 2 mg or IM 10 mg) Second dose after 5 min if administered IV If not effective, switch to status epilepticus protocol	Considering the high risk of transformation in status epilepticus in the case of recreational drug intoxication, watchful waiting during 5 min before administration of benzodiazepine is not recommended.
Status epilepticus	Benzodiazepine (midazolam 0.15 mg/kg IV) combined with levetiracetam 60 mg/kg IV over 10 min	If refractory: Therapeutic coma (e.g., propofol, midazolam).
Cerebral oedema	Manage hyponatremia: Strict fluid restriction (< 500 mL/24 h) 3% hypertonic saline for correction If intracranial hypertension: treat with mannitol	Often secondary to hyponatremia. Control natremia correction rate to prevent osmotic demyelination. Monitor neurological status closely. Preventive control of temperature and sodium balance is essential. Careful hydration (e.g., massive sympathomimetic states, hyponatremia)

## Data Availability

No new data were created or analyzed in this study. Data sharing is not applicable to this article.
